# A promising approach in laser vitrectomy executed by plasma-mediated removal of vitreous body via a diode-pumped Q-switched Nd:YAG laser

**DOI:** 10.1038/s41598-020-78878-y

**Published:** 2020-12-10

**Authors:** Daniel Uthoff, Rita Oravecz, Romina Kuehnl, Friederike Rubin-Schwarz, Matthias Frentzen, Norbert Schrage, Jörg Meister

**Affiliations:** 1 Aachen Center of Technology Transfer in Ophthalmology (ACTO E.V.), An-Institute RWTH Aachen University, Karlsburgweg 9, 52070 Aachen, Germany; 2 University of Applied Sciences Ansbach, Ansbach, Germany; 3 Institute of Biomedical Optics, University of Lübeck, Lübeck, Germany; 4 Department of Operative and Preventive Dentistry, Bonn University, Bonn, Germany; 5 Center of Dento-Maxillo-Facial Medicine, Bonn University, Bonn, Germany; 6 Center of Applied Medical Laser Research and Biomedical Optics (AMLaReBO), Bonn University, Bonn, Germany

**Keywords:** Applied physics, Biological physics, Fluid dynamics, Optical physics, Plasma physics, Techniques and instrumentation, Optics and photonics, Lasers, LEDs and light sources, Eye diseases, Retinal diseases, Vitreous detachment

## Abstract

To examine the applicability of plasma-mediated vitreous body removal, a diode-pumped Q-switched Neodymium:YAG laser was used for a possible application in eye surgery/vitrectomy. On a total of 1500 porcine vitreous bodies, removal rates were evaluated by comparing different LaserVit-tip designs (Mark I/II Gauge 19 and Mark III Gauge 22). The Nd:YAG laser, operating at a wavelength of 1064 nm and a pulse duration of 4 ns, was utilized for vitreous body removal with respective settings of 2, 3 and 4 mJ and pulse repetition rates (cut rates) from 5 to 25 Hz (300–1500 /min) in 5 Hz-steps as well as for 100 Hz (6000 cuts/min). The exposure times were selected at 10, 20, 40 and 60 s, respectively. Comparative measurements were carried out with mechanical cutters (Gauge 20 and Gauge 23), applying a fixed cut rate of 800 /min (13.33 Hz) at identical exposure times. The LaserVit-tips showed successful vitreous body removal for all laser settings and exposure times (Mark I: 6.2 g/min, Mark II: 8.2 g/min at 1500 cuts/min and 3 mJ, Mark II: 10.1 g/min, Mark III: 3.6 g/min at 6000 cuts/min at 3 mJ). Similar tip-dimensions (Gauge 22_laser_ and Gauge 23_cutter_) showed comparable removal rates of 3.6 g/min_laser_ and 1.3 g/min_cutter_ with settings of 6000 cuts/min at 3 mJ (laser) and 800 cuts/min for the mechanical cutter. A diode-pumped Q-switched Nd:YAG laser can successfully and gently remove vitreous body. The efficiency of the laser was comparable to that of mechanical cutters in terms of quantity of material removed per time unit.

## Introduction

Clouding or opacification of the vitreous body *(corpus vitreum)* of the eye, caused by—among other factors—traumatic effects of operations such as the intracapsular cataract extraction, has prompted various approaches for removing this gel-like substance. The first vitrectomies (vitreous body removal) were performed using swabs, scissors and tweezers. In the years 1966/1967, this procedure was successfully applied also in non-traumatized eyes, to remove *corpus vitreum* affected, for example, by secondary opacification from amyloidosis^1^. In the early 1970s^2^, Machemer et al. realized the first attempts of vitreous body removal by using rotating blades, followed by manual aspiration of the resulting vitreous body fragments. Subsequently, an infusion tube was additionally attached to the cutter mechanics (vitreous infusion suction cutter (VISC)) to prevent the bulbus from collapsing. With the introduction of the 3-port vitrectomy by O’Malley and Heintz, a technology was finally established which is still being employed today^3,4^. With regards to maximum efficiency and patient safety, there have since then been continuous advancements in the areas of cutter size, cut rate, blade design, port geometry and duty cycle^5^. Nowadays, there are 27-gauge vitrectomy probes available and cut rates of up to 16,000 cuts/min are possible. However, despite all these innovations, these systems are approaching their physical and mechanical limits.

Within the scope of these development phases, the traumatic stresses for the eyes have been continuously reduced, and the operations have become less invasive. Even so, the fact remains that the fragmentation of the vitreous body is based on the "Guillotine principle". As a result, the opening and closing of the aspirator port leads to adverse mechanical effects such as traction and shear forces within the vitreous body that are transferred to intraocular structures^6^.

A newly established approach for minimizing these stress factors is the liquefaction of the vitreous body by ultrasound. With frequencies in the lower kilohertz range (< 100 kHz), alternating stresses on the collagen- and hyaluronic acid matrix of the vitreous body are generated, thereby leading to liquefaction in a highly localized zone in front of the aperture. The first ophthalmological application of this particular technology was documented for phacoemulsification by Banko and Kelmann in 1971^7^. The successful fragmentation of vitreous bodies was demonstrated by Girard et al. [1976]^8^ and Leitgeb et al. 1979^9^. The further development of this technology led to the so-called "hypersonic-vitrector-systems"^10,11^.

Since the ultrasound-vitrectomy is an acoustics-based technology, possible advantages over classical mechanical (cutter) vitrectomy are obvious: There are no movable parts in the applicator and the aspirator port is always open, thereby minimizing adverse effects like traction and shear forces within the vitreous body. The extent to which vibrations of the applicator itself may exert mechanical effects on the retina is currently being studied.

Another alternative technology is the use of a laser system in vitrectomy. Comparable to ultrasound vitrectomy, laser vitrectomy also has no movable parts in the applicator and, thus, the aspirator port is always open. Around the turn of the millennium, the Er:YAG (Erbium-doped Yttrium Aluminum Garnet) laser was favored for laser vitrectomy because of its strong, energy absorption properties in water^12–15^. In vitro studies on open vitreous body were evaluated as promising^16^. In a comparative clinical study (erbium laser vs. mechanical cutter), Petersen et al. documented a reduced traumatic stress on the retina by using the Er:YAG laser^17^. At the same time, Binder et al. obtained comparable results in another clinical study^18^. In addition, both author groups reported no thermal effects during the treatment.

Caused by the high absorption of energy at the wavelength of 2.94 µm (Er:YAG) in water, cavitation bubbles arise, which raises questions about the benefits of this technology. The expansion of cavitation bubbles always results in a displacement of the material that has to be handled (independently whether it is in liquid- or gel-like form). The shock waves that occur during the oscillation as well as collapse of the cavitation bubble may cause undesired mechanical damage in the surrounding tissue structures. Moreover, since such jet-like streams from the aspiration opening reduce the volume of aspirated vitreous, the removal efficiency is essentially impaired^19,20^. Thus, related influencing parameters such as pulse energy, pulse repetition rate, pulse duration and applicator design were the topics investigated by diverse studies^21^. For vitrectomy, this basically implies a limitation of the removal rates up to 0.07 g/min^21^, independently of the design of the aspiration opening (open-end or side-firing) which were confirmed in (unpublished) preliminary investigations to this current work.

Therefore, within the scope of this current study, the Q-switched Nd:YAG (Neodymium-doped Yttrium Aluminum Garnet) laser was used as an alternative to the Er:YAG laser for the plasma-mediated laser vitrectomy. The Nd:YAG radiation is absorbed by a titanium surface within the handpiece, which ignites a plasma and generates a shock wave that enables tissue destruction. By using high pulse repetition rates at given low pulse energies, the surgeon may be able to perform vitreoretinal operations with vitreous body removal that is efficient and less invasive for the patient. Consequently, the aim of this current study is to evaluate the feasibility of vitreous removal applying a diode-pumped Q-switched Nd:YAG laser, based on the removal rates of porcine vitreous body.

## Materials and methods

### Ethical approval

Eyes were explanted *postmortem* by a slaughterhouse approved according to the guidelines of the German Regulations for Animal Protection and Slaughter, issued by the German Federal Ministry for Consumer Protection, Food and Agriculture in 2012.

### Sample preparation

Vitreous body from freshly slaughtered pigs (porcine model: German Landrace swine, 8 month old, mass 30–40 kg) was used for laser vitrectomy. The vitreous bodies of the pigs were surgically extracted from every pig eye within 1 up to 4 h postmortem. A total of 1500 pig eyes from 750 animals (two specimens from each animal) were used. Experimental investigations were carried out directly after the eyes were present in the lab (1–4 h postmortem).

### Laser source

**Figure 1:**
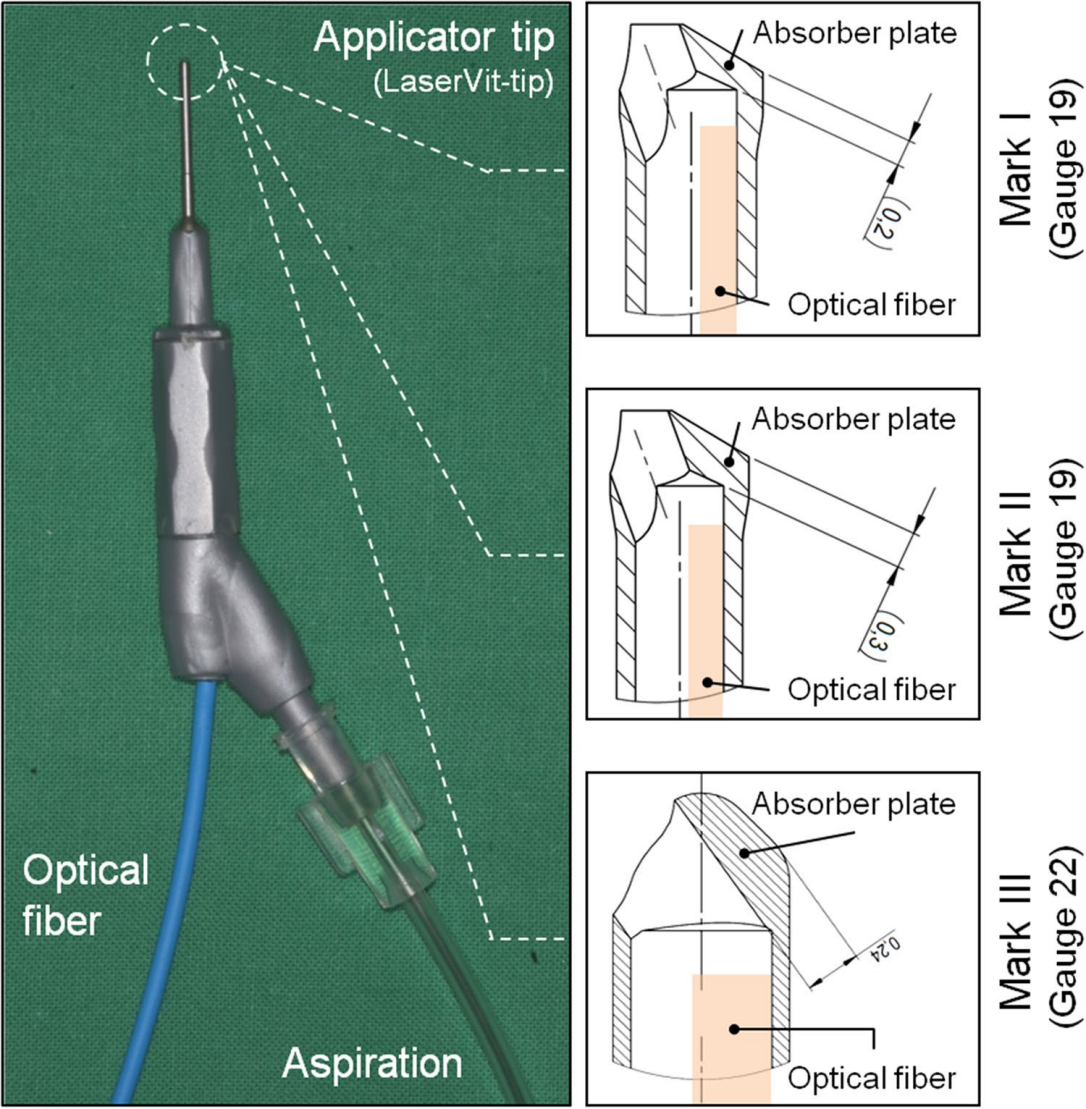
Ophthalmological handpiece used for vitreous body removal. An overview is shown on the left-hand side of the figure. The optical fiber as well as the aspiration tube is adapted to the handpiece. The applicator (LaserVit-tip) is at the end of the handpiece. Technical drawings of the different LaserVit-tip designs (Mark I-III) are shown on the right-hand side of the figure. Technical details are listed in Table 1.

A diode-pumped Q-switched Neodymium:YAG laser (λ = 1064 nm, *A.R.C. Laser GmbH, Nuremberg, Germany*) was used for this investigation. The given pulse duration was 4 ns. Pulse energies of 2, 3 and 4 mJ, respectively, were chosen for irradiation. Selected pulse repetition rates were 5 Hz up to 25 Hz at increments of 5 Hz, and an additional frequency of 100 Hz was applied. Laser light was transmitted to the handpiece (Fig. 1) by using an adapted 320 µm-diameter quartz fiber (NA = 0.22).

For aspiration experiments of the vitreous body, handpieces with newly designed titanium applicator tips (LaserVit-tip, *A.R.C. Laser GmbH, Nuremberg, Germany*) were utilized in a stepwise modification (Mark I – III) during the investigations (Fig. 1 and Table 1). Aspiration was carried out with a Phaco machine (*Megatron, Geuder AG, Heidelberg, Germany*). The chosen settings were 550 mmHg for the vacuum, a flow rate of 50 ml/min and ramp time of 0.5 s for the Venturi effect.

**Table 1:** Data and remarks about the LaserVit-tips (Mark I-III) used for vitreous body removal.

Type:	Mark I	Mark II	Mark III
Position Fig. 1:	Right side: top	Right side: center	Right side: bottom
Size^22^:	Gauge 19	Gauge 19	Gauge 22
Dimensions /mm:	∅_outer_ = 1.06, ∅_inner_ = 0.7	∅_outer_ = 1.06, ∅_inner_ = 0.7	∅_outer_ = 0.76, ∅_inner_ = 0.6*
Remarks:	Absorber plate thickness within the laser interaction zone is 0.2 mm	Absorber plate thickness within the laser interaction zone is 0.3 mm	Absorber plate thickness within the laser interaction zone is 0.24 mm

*Customized.

### Experimental setup (laser-induced aspiration)

For these in vitro experiments, only the removal of the vitreous body was of interest. No irrigation was applied. Every single measurement was performed with new vitreous bodies and new handpieces. To determine the influence of the different LaserVit-tips (Mark I – III) regarding aspiration, suction rates were recorded at 10, 20, 40 and 60 s, respectively. Furthermore, the mass difference was determined by using an electronic balance (*SBS-LW-2000A, Steinberg Systems, Zielona Góra, Poland*) before and after removal.

Depending on the suction time, 4 up to 7 vitreous bodies were used per measurement (mass of one porcine vitreous was approx. ≈ 2.9 ± 0.4 g). After removal from the pig eye, the vitreous bodies were collected in a beaker. The handpieces were positioned approximately 5 mm above the bottom of the beaker.

### Experimental setup (mechanical-induced aspiration)

The same aforementioned experimental conditions regarding the vitreous bodies were retained for standard guillotine driven mechanical-induced “cutter” removal. For this purpose, a Phaco machine (*Megatron, Geuder AG, Heidelberg, Germany*) was used. The chosen settings were adapted to the laser-induced aspiration, 550 mmHg for the vacuum, a flow rate of 50 ml/min and a ramp time of 0.5 s for the Venturi effect. The mechanical handpiece sizes were Gauge 20 and Gauge 23, the cut rate was 800/min (13.33 Hz) and the measuring times were 10, 20, 30, 40 and 60 s, respectively.

The same aspiration experiments were carried out with and without laser activity using pure water as a reference material.

### Statistical evaluation

**Figure 2:**
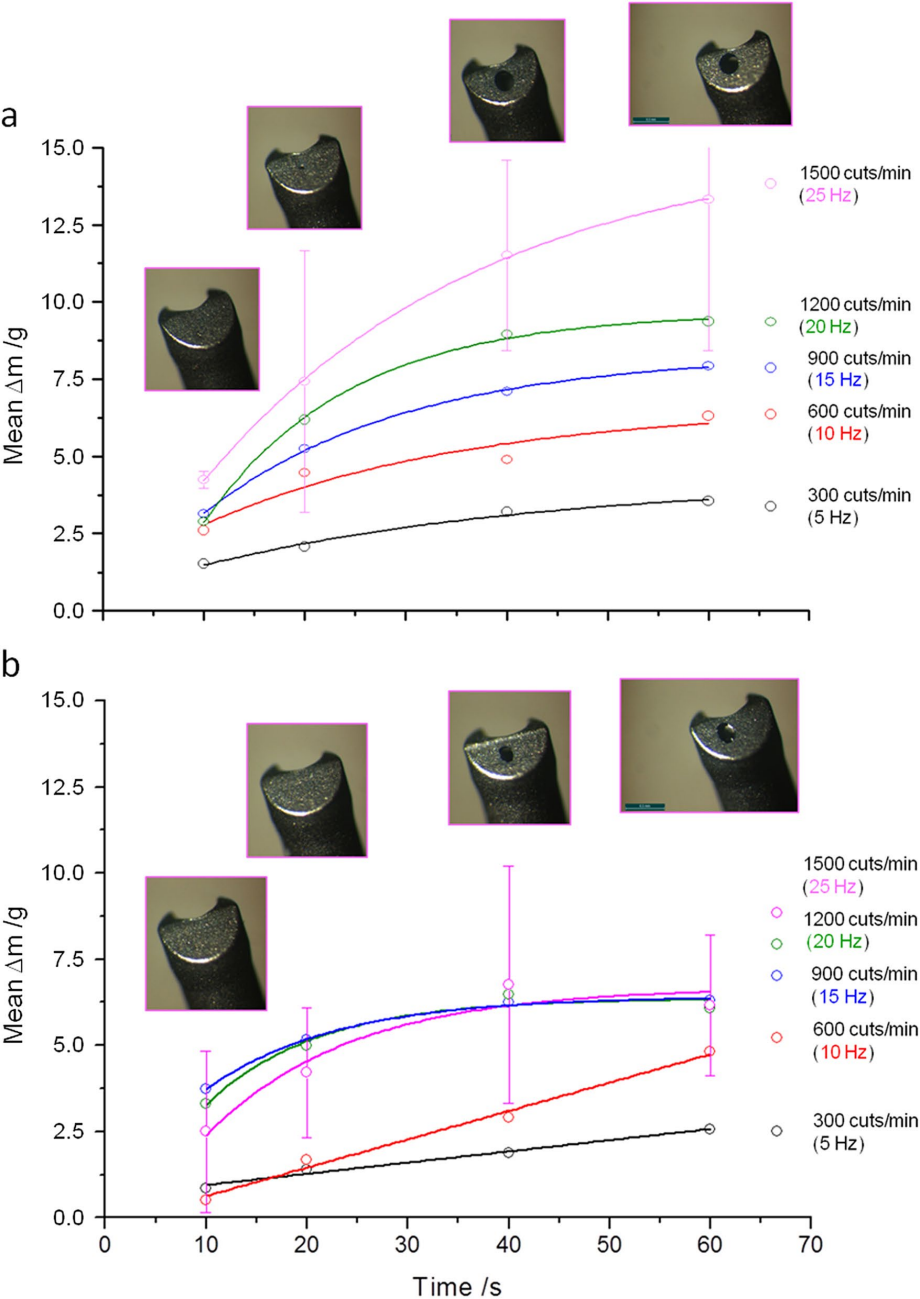
Aspiration rates given for the Mark I handpiece at 4 mJ (2a) and 3 mJ (2b) pulse energy and cut rates of between 300–1500 cuts/min (5–25 Hz). Above the 25 Hz graph are images of the absorber plates located at the distal end of the LaserVit-tips. By exceeding measurement times of ≥ 40 s, absorber plates are abraded by laser radiation.

**Figure 3:**
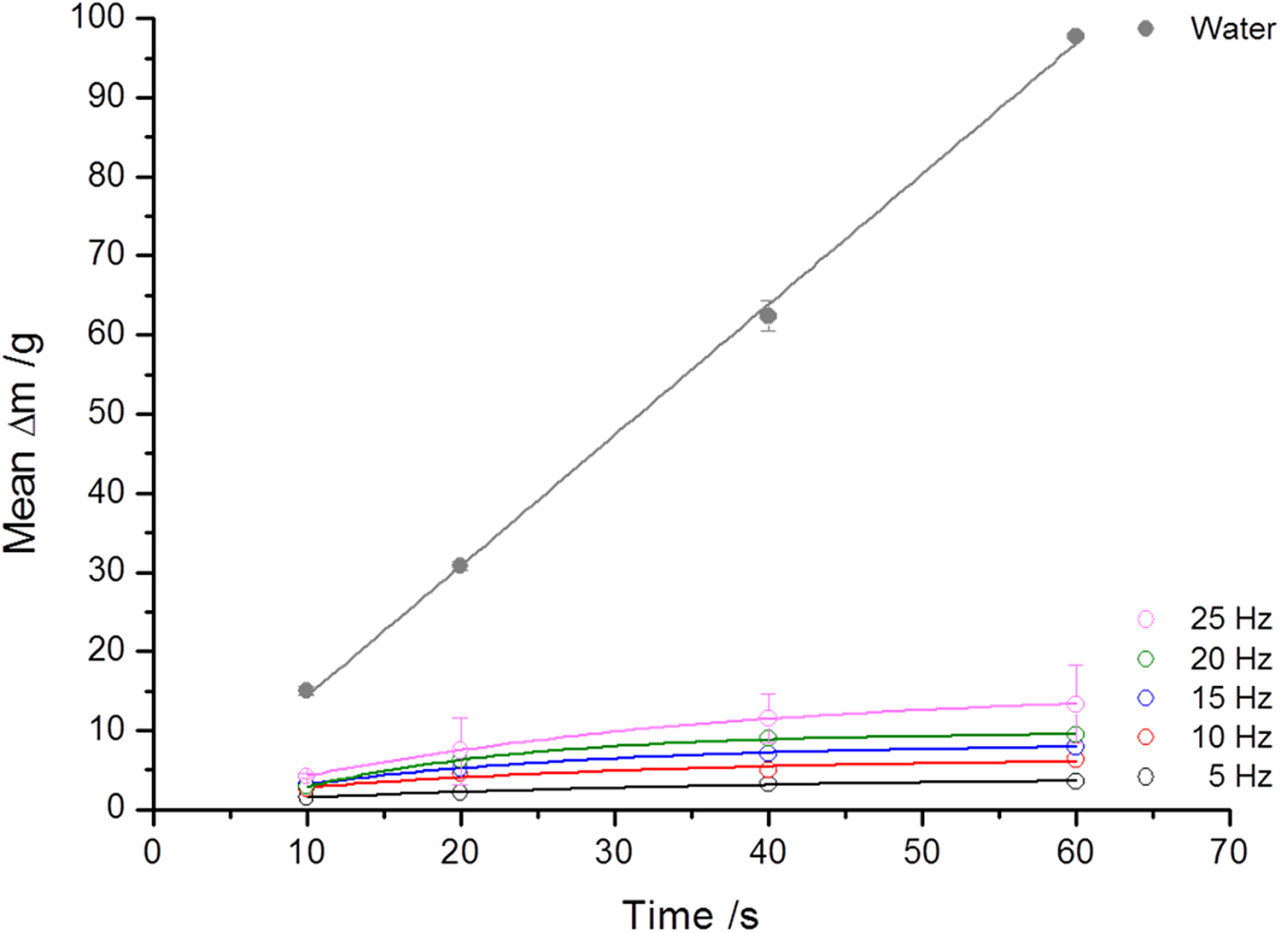
Using the example of Fig. 2a, a comparison of the results of porcine vitreous body aspiration with that of water as reference material using Mark I handpieces. The difference in the aspiration rate is approximately one order of magnitude. Corresponding cut rates are also given in Fig. 2a.

Descriptive evaluation, including calculation of the mean values, standard deviations and error estimations were applied to show the principle functioning of the LaserVit-tips. The spreadsheet calculation program OriginPro 8G (OriginLab Corporation, Northampton, MA, USA) was used for data evaluation of the vitreous mass removal. Graphical evaluation of the results was carried out as well with OriginPro 8G. Exponential and linear fit functions were applied in Figs. 2 and 3 as well as in Figs. 5, 6 and 7 for visualizing the data behaviors (trend curves). An explorative statistical analysis was not yet appropriate.

## Results

### Mark I handpiece

By means of the first aspiration tests, the framework conditions were investigated whether this method principally meets the requirements for vitreous body removal. The pulse energies used here were 4 mJ und 3 mJ with cut rates ranging from 300 to 1500 cuts/min (corresponding to pulse repetition rates of 5–25 Hz). Figure 2a,b show the attained removal rates for pulse energies of 4 mJ and 3 mJ, respectively.

The increase in the aspiration rates correlates with the increase in the pulse energy, the pulse repetition rate and the irradiation time. The maximally attained aspiration rates at 1500 cuts/min (25 Hz und 60 s) were: 13.3 g/min for E_4 mJ_, and 6.2 g/min for E_3 mJ_. For cut rates of ≥ 600 cuts/min (10 Hz), a decrease in the slope of the curves can be observed for both pulse energies, as well as a saturation behavior at a pulse energy of 3 mJ. Due to the enormous fluctuation margin of the determined aspiration rates, the standard deviation values are reported for pulse energies of 4 mJ and 3 mJ, exemplary for the 25 Hz measurements. The standard deviation values for all other measurement values can be represented in the same order of magnitude.

By directly comparing Fig. 2a,b, the difference between both pulse energies as well as between the reference liquid water can be seen (Fig. 3). Here, water shows (independently of whether with or without laser activity) a linear increase over the entire timeline of 60 s with attained aspiration rates of 95.4 g/min.

**Figure 4:**
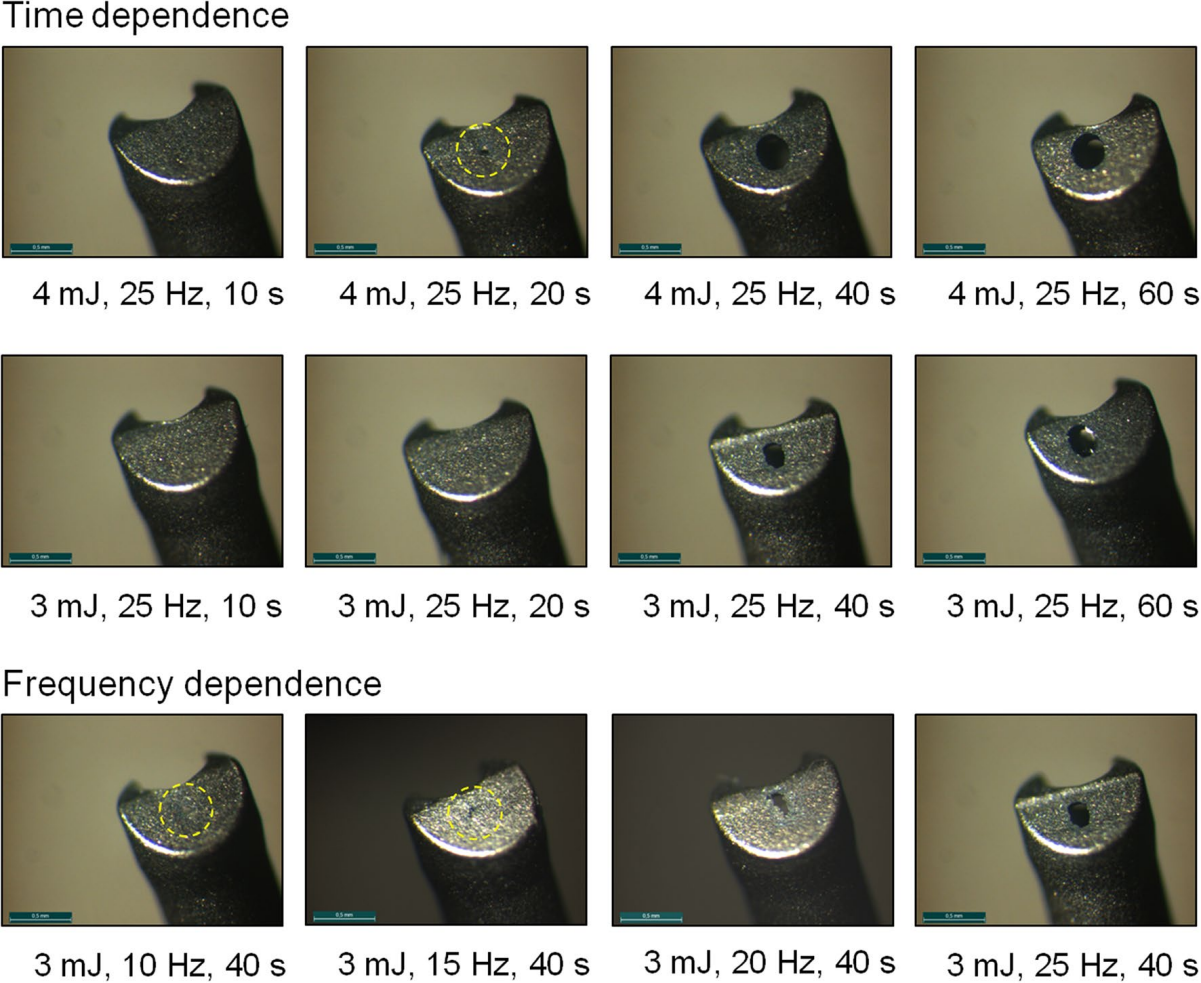
Absorber plates of Mark I handpieces in time-dependence [3 mJ and 4 mJ at 1500 cuts/min (25 Hz)] and frequency-dependence [3 mJ and 40 s in a range of 600–1500 cuts/min (10–25 Hz)]. The interaction between the absorber plate and laser radiation (plasma formation) is needed for vitreous body cutting to ensure aspiration. The destruction of the absorber plates affected the cutting process, resulting in a reduced slope of the curve at ≥ 600 cuts/min (10 Hz) and therefore in a reduction of the aspiration rate (Fig. 2a,b).

Furthermore, Fig. 2a,b show the distal end of the LaserVit-tips for the respective maximum cut rate after ending the measurement. At irradiation times of ≥ 40 s, there was a definite abrasion for both pulse energies observed at the absorber plate material in the application tip. Not only was this material destruction associated with increasing irradiation time, but also with increasing laser frequency (Fig. 4). Depending on the exposure time, the penetration through the absorber plate begins already after 20 s at a pulse energy of 4 mJ. At a pulse energy of 3 mJ, however, this effect appears later (> 20 s). Depending on the frequency, at a fixed pulse energy (3 mJ) and an irradiation time of 40 s, a material abrasion is shown from the very beginning and increases with the cut rate and, thus, with the pulse repetition rate.

The destructive processes, that testifies an indirect proof of plasma formation occurring in the LaserVit-tips, highlight the necessity to modify the stability, which was technically implemented in the Mark II handpiece by using a thicker material layer in the absorber plate (Fig. 1).

**Figure 5:**
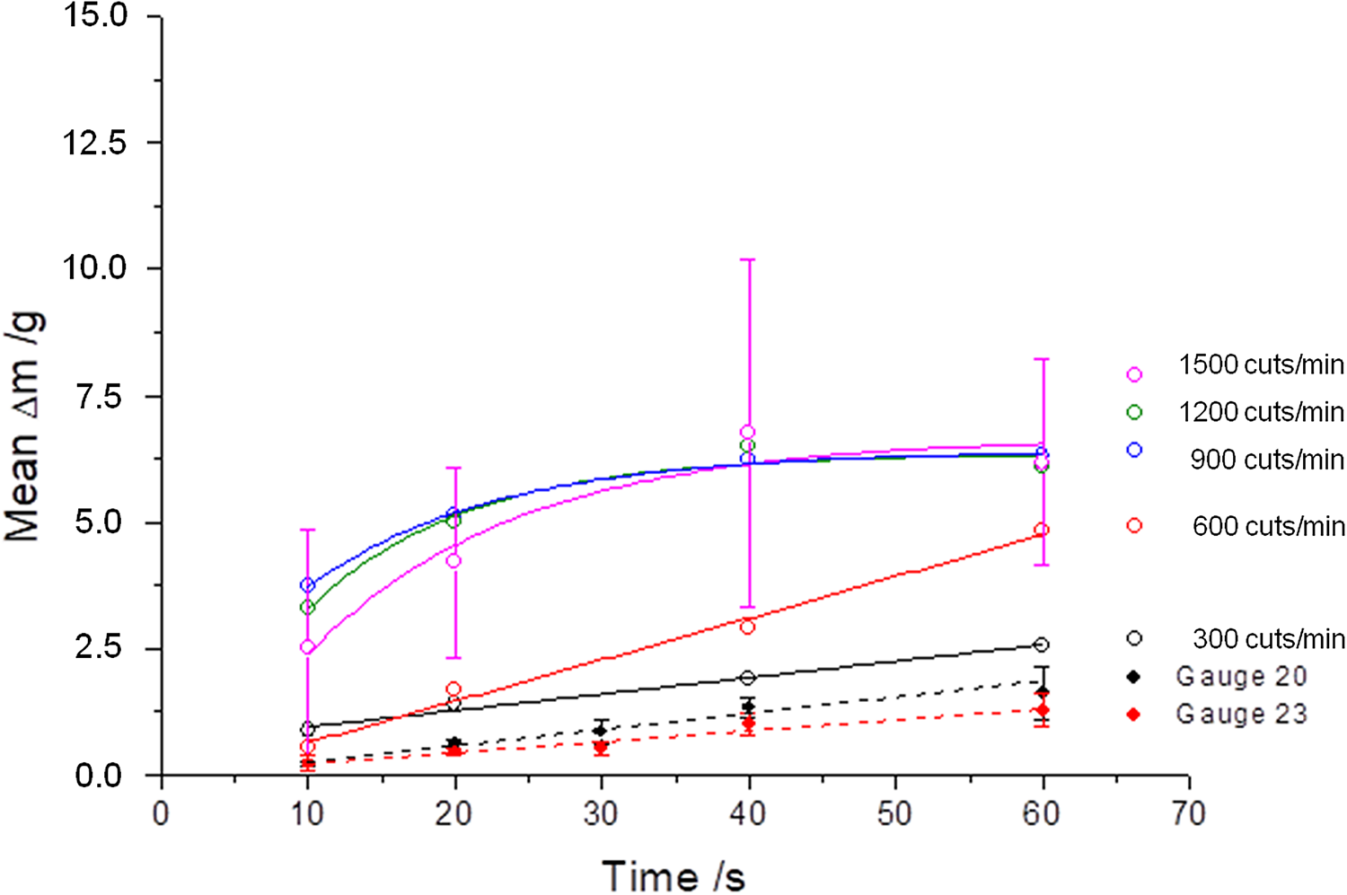
Aspiration rates using a Mark I handpiece (Gauge 19) at a pulse energy of 3 mJ (Fig. 2b) compared to a mechanical cutter with two different applicator tips [Gauge 20 and 23 at 800 cuts/min (13.33 Hz)] as a clinical reference. Due to the low aspiration rates of the mechanical cutter, only pulse energies of ≤ 3 mJ were used for Mark II and III handpieces.

Furthermore, a comparative measurement was undertaken for estimating the status quo of the laser-induced aspiration in relation to the clinically applied cutters. Figure 5 illustrates the obtained aspiration rates of the mechanical system with two different applicators (Gauge 20 and Gauge 23), in comparison with the laser-induced aspiration at a pulse energy of 3 mJ using a Mark I-LaserVit-type (Fig. 2b).

Similar to the laser-induced aspirations in the lower cut-region, the aspiration rates of the mechanical cutter showed a linear increase with time. Due to the low aspiration rates of 1.3 g/min_(Gauge 23)_ < 1.6 g/min_(Gauge 20)_ <  < E_3 mJ_ of 6.2 g/min_(Gauge 19)_ at 900 cuts/min (15 Hz), measurements with a pulse energy of 4 mJ were waived for further studies with the Mark II handpiece.

### Mark II handpiece

**Figure 6:**
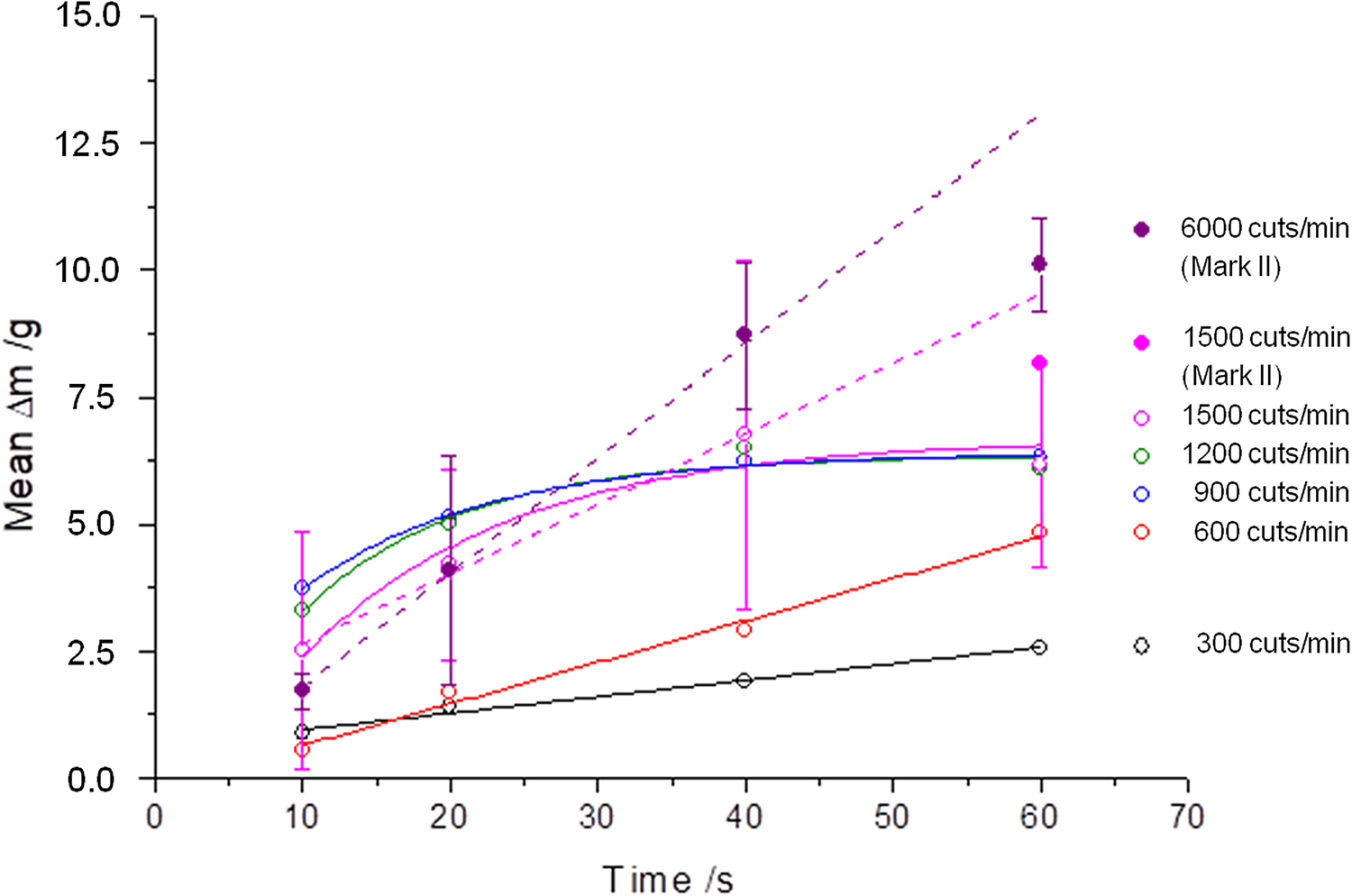
The technological progress of the new handpiece Mark II is shown by the increase in the aspiration rate at 1500 cuts/min and 60 s irradiation time. Furthermore, 6000 cuts/min are now feasible.

In contrast to the Mark I LaserVit-tips, the Mark II LaserVit-tips have a thicker material layer (0.3 mm) in the absorber plate (Table 1 and Fig. 1). The effect is clearly shown by the decrease in the slope, i.e. by an increase in the aspiration rate from 6.3 to 8.2 g/min at a pulse energy of 3 mJ and a cut rate of 1500 cuts/min (25 Hz). The increase in the cut rate to 6000 cuts/min (100 Hz) rate revealed an aspiration rate of 10.1 g/min. With this improvement, for the first time a linear increase in the aspiration rates could be shown at high pulse repetition rates of 1500 cuts/min and 6000 cuts/min up to 40 s irradiation time (Fig. 6).

**Figure 7:**
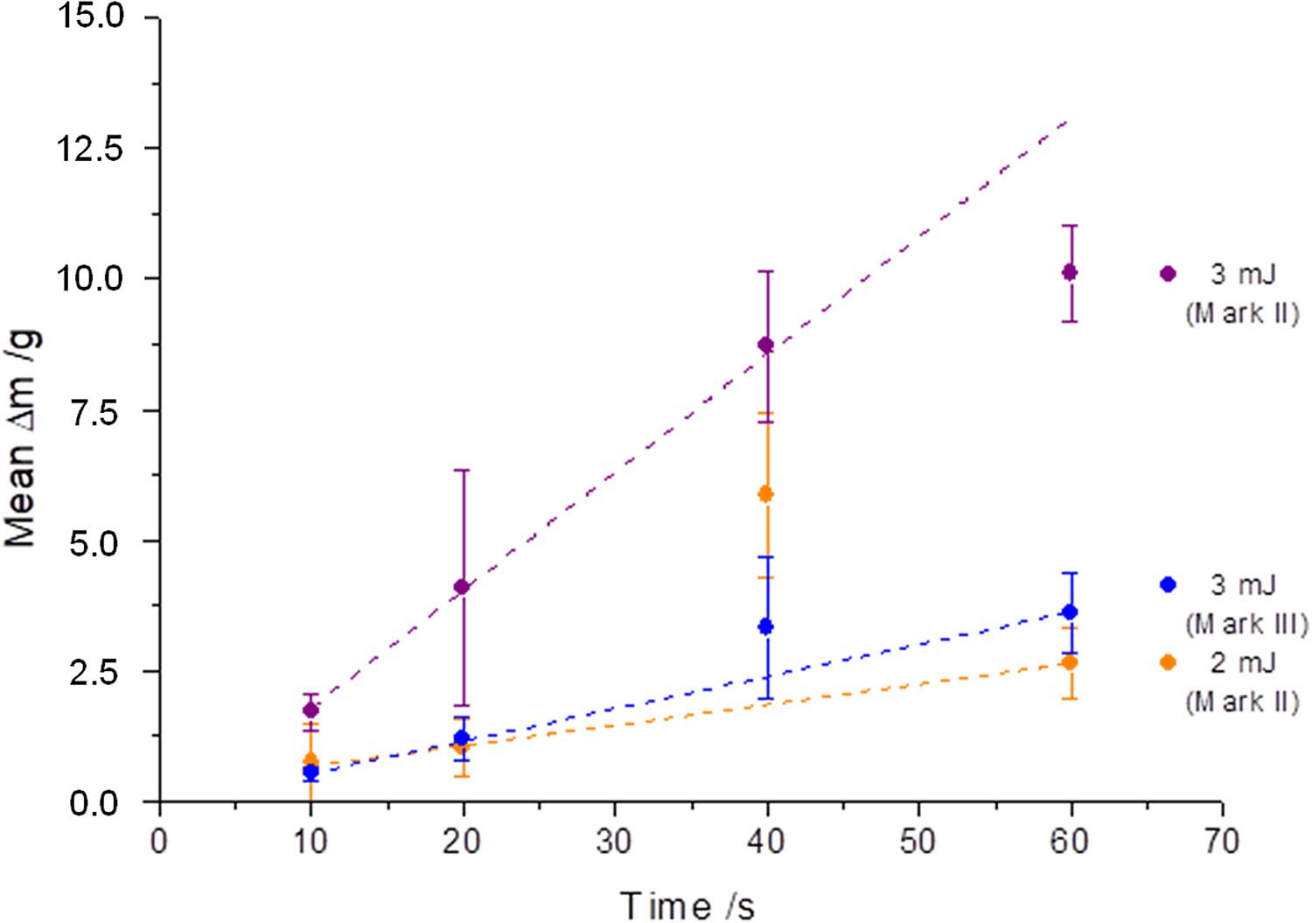
Mark II and Mark III handpieces at pulse energies of 2 mJ and 3 mJ at 6000 cuts/min (100 Hz) for all measurements. Comparable results were achieved for Mark II at 2 mJ and Mark III at 3 mJ.

With potential clinical application in mind, the pulse energy was reduced to 2 mJ. This step reduced the aspiration rate to 2.6 g/min (Fig. 7) which, however, still exceeded the aspiration rates of the mechanical cutter (Fig. 5).

### Mark III handpiece

By reconsidering the clinical application, the diameter of the handpiece (Mark III) was reduced to Gauge 22 (Table 1 and Fig. 1). At a pulse energy of 3 mJ and a cut rate of 6000 cuts/min (100 Hz), the maximum aspiration rate of 3.6 g/min was reached (Fig. 7). Until now, no clinically relevant aspiration rates could be obtained for the Mark III handpiece by reducing once again the pulse energy to 2 mJ.

## Discussion

### Method (choice of Q-switched Nd:YAG)

Different methods were carried out for vitreous body removal. Regardless of the different techniques used, the material destruction of vitreous body is required because of its viscosity properties and anatomical structure. The underlying problem, however, is the fact that the employed handpieces need to have sizes of Gauge ≥ 22 (∅ ≤ 0.76) in order to minimize surgical side effects. Therefore, independently of the techniques applied, the aspiration of vitreous body via capillaries always poses a technical challenge.

The advantage of the Nd:YAG laser lies in the high transparency of light at the wavelength of 1.064 µm in water (µ_A_ = 0.363 cm^−1^)^12^. Only by using pulse durations in the nanosecond-range and focusing the radiation, these laser systems can generate optical breakdowns, accompanied by generating cavitation bubbles. Contradictory statements have been made about the rheology of *corpus vitreum* in the literature: Whereas Krauss et al. found no essential changes of the vitreous body^23^ after Q-switch Nd:YAG laser irradiation, Abdelkawi et al. could determine clear effects on vitreous body rheology (e.g., a change in protein content, refractive index, viscosity and consistency)^24,25^.

With regard to the technical implementation of the vitreous body processing in this current work, a 2-step interaction could be employed as the cutting process. Due to the use of a quartz fiber for transmitting light into the ophthalmological handpiece, and because of its size, no additional internal focusing optics could be utilized here. The combination of the design of the LaserVit-tips (side aspiration port) with the positioning of the optical fiber at a distance of 1 mm up to 2 mm from the front of the titanium absorber, generated a primary interaction with an accompanying plasma- and shock wave propagation (1st Step). Based on the fact that the refractive indices of fused silica and vitreous humor are approximated (n_fused silica_ = 1.449@1.083 µm, n_vitreous humor_ = 1.341@650 nm)^26,27^, the generated intensity of the emitted Nd:YAG laser light of 3.1⋅10^8^ W cm^−2^ is in the order of magnitude of the titanium absorber threshold^28^. Plasma- and shock waves in this technique tear the vitreous body inside the LaserVIT-tip so that the vitreous body can be aspirated (2nd Step).

### Vitreous body

The vitreous body is a clear transparent hydrogel in various species and consists, more or less, of identical extracellular matrix components. It contains a very high amount of water (98–99%) and traces of hyaluronic acid, glucose, ions and collagen [(11–16)⋅10^−3^%)^29–31^. Hyaluronic acid and collagen are the major components of the fiber matrix and responsible for the gel-like structure of the vitreous body; these components are, however, not homogeneously distributed^32^. The vitreous body is classified as a viscoelastic material and exhibits non-Newtonian rheological properties^32–34^, stemming from the varying concentrations of hyaluronic acid and collagen in the anterior, central and posterior regions^35^. Moreover, differences in the matrix composition mentioned above are responsible for the particular rheologic state of the vitreous in different species^36^.

Despite the fact that the vitreous body exhibits rheological properties like a non-Newtonian fluid^23–25^, values of the viscosity are not clearly defined in the literature. Some data are given as dynamic viscosity adopted to Newton’s law as a Newtonian fluid, given in Eq. (1):

1 $$\upeta =\upnu \cdot\uprho, $$

whereby η denotes the dynamic viscosity, ν the kinematic viscosity and ρ the density of the substance. Other data are given as apparent viscosities adopted to non-Newtonian fluids. In that case, the viscosity η depends additionally on the shear rate δ or the shear stress σ^34^. This involves a spatial separation of the viscosity measurements within the anterior, central and posterior regions of the vitreous body^32,37,39^. Therefore, the inhomogeneous viscosity could explain the erratic values (see error bars) shown in Fig. 2a,b at 1500 cuts/min (25 Hz).

Compared to the dynamic viscosity of water (1.0087 mPa⋅s at 20 °C), the viscosity of porcine vitreous body is much higher (Table 2). The resulting effects are reflected in the differences in the aspiration rates shown in Fig. 3. Regarding the clinical applications, the viscosity of human vitreous body is approximately one order of magnitude lower than porcine vitreous body. Therefore, higher aspiration rates can be expected along with smaller handpieces (Gauge 22) and lower pulse energies (2 mJ).

**Table 2:** Viscosities of human and porcine vitreous body from the literature.

Literature	Human	Porcine
Lee et al.^32^	1.4–4.9 Pa⋅s*	–
Kawano et al.^34^	0.38–0.62 Pa⋅s**	0.0026–0.003 Pa⋅s*
Lee et al.^37^	–	1.8–12.1 Pa⋅s*
Pokki et al.^38^	3.6 ± 0.9 Pa⋅s	40.4 ± 14.5 Pa⋅s
Donati et al.^39^	0.3–2.0 Pa⋅s	–
Chirila and Hong^#^^40^	0.0016 Pa⋅s**	–

*Apparent viscosity: The vitreous body exhibits non-Newtonian rheological properties based on spatially varying concentrations of hyaluronic acid and collagen^32,34,37^.

**Dynamic viscosity: The vitreous body exhibits rheological properties as a Newtonian fluid adopted to Newtons law^34,40^.

^#^Values from^40^ describe the viscosity of "aspirated" human vitreous body (Reference by Shafer^41^).

### LaserVit-tips–handpiece design

The objective of further developments of the LaserVit-tips is to implement a standardized application size of Gauge 23 for clinical use. Nevertheless, it is not only the size but also the lifetime and aspiration rate, i.e. the mass of vitreous body removed per time unit, that are decisive for a gentle, relatively non-invasive therapy, that likewise saves time. Based on the complex viscosity properties of the vitreous body, the ratio of the inner diameter of the LaserVit-tip (Gauge 23: ∅_outer_ = 0.68 mm, ∅_inner_ = 0.4 mm_customized_) to the optical fiber diameter (∅_outer_ ≤ 0.28 mm) is particularly important for a gentle and simultaneously time-saving therapy. In order to create suitable aspiration conditions, the ratio ∅_inner, tip_/∅_outer, fiber_ should lie in the magnitude of ≥ 2:1^42^.

However, reducing the optical fiber diameter causes higher intensities on the absorber plate which, in turn, reduces the lifetime of the LaserVit-tip. This can be compensated by a reduction in the pulse energy of the laser. Ideally, the extent to which these ideas can mesh with one another is the aim of future investigations.

## Conclusions

Within the scope of this study, it was possible for the first time to technically realize the efficient removal of vitreous body using a diode-pumped Q-switched Nd:YAG laser. The plasma-mediated laser vitrectomy represents a new and promising approach in this regard. The results show that laser-induced aspiration rates comparable to those of mechanical cutters could be obtained. However, for clinical applications and use, further investigations are necessary with respect to handpiece size, tip-design as well as optimized laser parameters. Nevertheless, a transfer of this laser technology into the area of clinical ophthalmology is feasible. Based on these promising results, this technology is approved for testing in animal experiments on rabbit eyes.

We strongly believe that a new branch has opend up for vitrectomy. Modifications of the LaserVit-tips enable an additional illumination and a variability in geometry. With a clinical application in mind even a one-handed vitrectomy is conceivable.
